# Accuracy and precision of volumetric bone mineral density assessment using dual-source dual-energy *versus* quantitative CT: a phantom study

**DOI:** 10.1186/s41747-021-00241-1

**Published:** 2021-10-05

**Authors:** Vitali Koch, Nils Große Hokamp, Moritz H. Albrecht, Leon D. Gruenewald, Ibrahim Yel, Jan Borggrefe, Stefan Wesarg, Katrin Eichler, Iris Burck, Tatjana Gruber-Rouh, Lukas Lenga, Thomas J. Vogl, Simon S. Martin, Julian L. Wichmann, Renate M. Hammerstingl, Leona S. Alizadeh, Christoph Mader, Nicole A. Huizinga, Tommaso D’Angelo, Giorgio Ascenti, Silvio Mazziotti, Christian Booz

**Affiliations:** 1 Division of Experimental Imaging, Department of Diagnostic and Interventional Radiology, University Hospital Frankfurt, Theodor-Stern-Kai 7, 60590 Frankfurt am Main, Germany; 2 Department of Diagnostic and Interventional Radiology, University Hospital Cologne, Cologne, Germany; 3 Department of Radiology, Neuroradiology and Nuclear Medicine, Minden Hospital, University of Kiel, Kiel, Germany; 4 Cognitive Computing and Medical Imaging, Fraunhofer IGD, Darmstadt, Germany; 5 Department of Diagnostic and Interventional Radiology, University Hospital Frankfurt, Frankfurt am Main, Germany; 6 Interdisciplinary Center for Neuroscience, Goethe University of Frankfurt, Frankfurt am Main, Germany; 7 Department of Biomedical Sciences and Morphological and Functional Imaging, University Hospital Messina, Messina, Italy

**Keywords:** Bone density, Dual-energy computed tomography, Osteoporosis, Phantoms (imaging), Tomography (x-ray computed)

## Abstract

**Background:**

Dual-source dual-energy computed tomography (DECT) offers the potential for opportunistic osteoporosis screening by enabling phantomless bone mineral density (BMD) quantification. This study sought to assess the accuracy and precision of volumetric BMD measurement using dual-source DECT in comparison to quantitative CT (QCT).

**Methods:**

A validated spine phantom consisting of three lumbar vertebra equivalents with 50 (L1), 100 (L2), and 200 mg/cm^3^ (L3) calcium hydroxyapatite (HA) concentrations was scanned employing third-generation dual-source DECT and QCT. While BMD assessment based on QCT required an additional standardised bone density calibration phantom, the DECT technique operated by using a dedicated postprocessing software based on material decomposition without requiring calibration phantoms. Accuracy and precision of both modalities were compared by calculating measurement errors. In addition, correlation and agreement analyses were performed using Pearson correlation, linear regression, and Bland-Altman plots.

**Results:**

DECT-derived BMD values differed significantly from those obtained by QCT (*p* < 0.001) and were found to be closer to true HA concentrations. Relative measurement errors were significantly smaller for DECT in comparison to QCT (L1, 0.94% *versus* 9.68%; L2, 0.28% *versus* 5.74%; L3, 0.24% *versus* 3.67%, respectively). DECT demonstrated better BMD measurement repeatability compared to QCT (coefficient of variance < 4.29% for DECT, < 6.74% for QCT). Both methods correlated well to each other (*r* = 0.9993; 95% confidence interval 0.9984–0.9997; *p* < 0.001) and revealed substantial agreement in Bland-Altman plots.

**Conclusions:**

Phantomless dual-source DECT-based BMD assessment of lumbar vertebra equivalents using material decomposition showed higher diagnostic accuracy compared to QCT.

## Key points

- Dual-source dual-energy CT (DECT) allowed for accurate bone mineral density (BMD) assessment.
- DECT-based BMD measurements showed a better repeatability in comparison to quantitative CT.
- DECT enables viable retrospective volumetric BMD measurements without requiring calibration phantoms.

## Background

Osteoporosis represents the most common metabolic bone disease characterised by decreased bone mineral density (BMD) and elevated fracture risk [1, 2]. Demographic changes with concomitant increase in osteoporosis entail substantial socioeconomic burden [1].

According to the World Health Organization, dual-energy x-ray absorptiometry (DXA) is the current gold standard for BMD assessment due to widespread availability and cost-effectiveness [3]. However, numerous limitations of DXA have been reported, such as the inability to differentiate between cortical and trabecular bone and distortion of BMD measurement through overlying structures [4–8]. Quantitative computed tomography (QCT), the current clinical standard for volumetric BMD assessment, is limitedly available due to high radiation exposure and the need for in-scan calibration using phantoms that do not represent the true composition of trabecular bone and prevent retrospective opportunistic BMD assessment in routinely performed computed tomography (CT) scans [9–11].

Given the increasing number of CT scans in recent years, retrospective BMD assessment by measurement of trabecular Hounsfield units (HU) has been repeatedly suggested, despite being associated with inaccuracies due to inhomogeneous trabecular bone composition as well as technical aspects such as variation of tube voltages [12–15].

Dual-energy CT (DECT) allows for improved material differentiation by using energy dependence of the photoelectric effect at different x-ray spectra [16]. This technique has provided novel and clinically relevant information regarding various musculoskeletal applications compared to conventional CT [17, 18]. Previously, a phantomless dual-source DECT postprocessing algorithm which enables volumetric opportunistic BMD assessment of lumbar trabecular bone by application of dedicated material decomposition has been reported [16]. Initial studies have shown promising results both *in vivo* compared with DXA and *in vitro* compared with pull-out-forces in human cadaver vertebra specimens [19, 20]. In addition, this algorithm yielded significantly more accurate BMD assessment of the lumbar spine and superior diagnostic accuracy for the detection of osteoporosis compared to HU measurements in a recently published study [21]. However, the accuracy and precision of dual-source DECT has not yet been compared to QCT [22].

We hypothesised that volumetric dual-source DECT using material decomposition may yield more accurate and precise volumetric BMD assessment compared to QCT and facilitates osteoporosis screening by enabling retrospective opportunistic BMD measurements in routine CT scans without the need for calibration phantoms. Thus, the purpose of this study was to compare the accuracy and precision of phantomless dual-source DECT and QCT for volumetric BMD assessment in a prospective phantom study.

## Methods

This prospective phantom study did not need any approval by the institutional review board.

### Phantoms

**Fig. 1:**
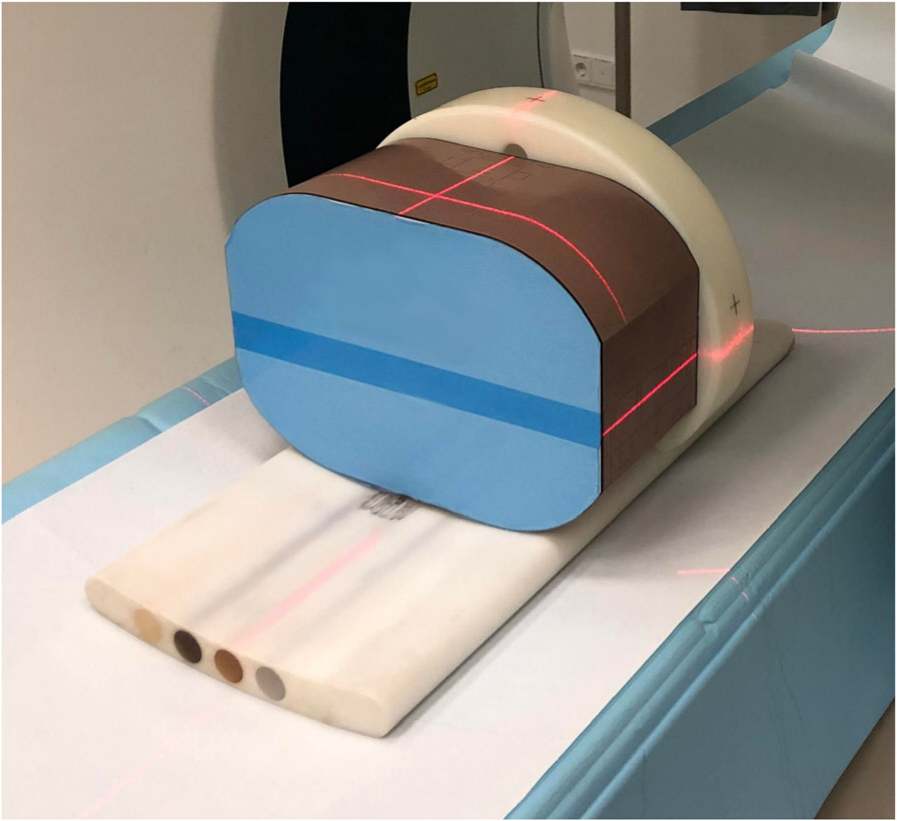
Illustration of the European spine phantom (blue, brown) (ESP-040; QRM GmbH, Moehrendorf, Germany) and the QCT bone density calibration phantom (white, below) (BDC-03-29; QRM GmbH, Moehrendorf, Germany). Labels of the manufacturers have been removed. QCT, Quantitative computed tomography

**Fig. 2:**
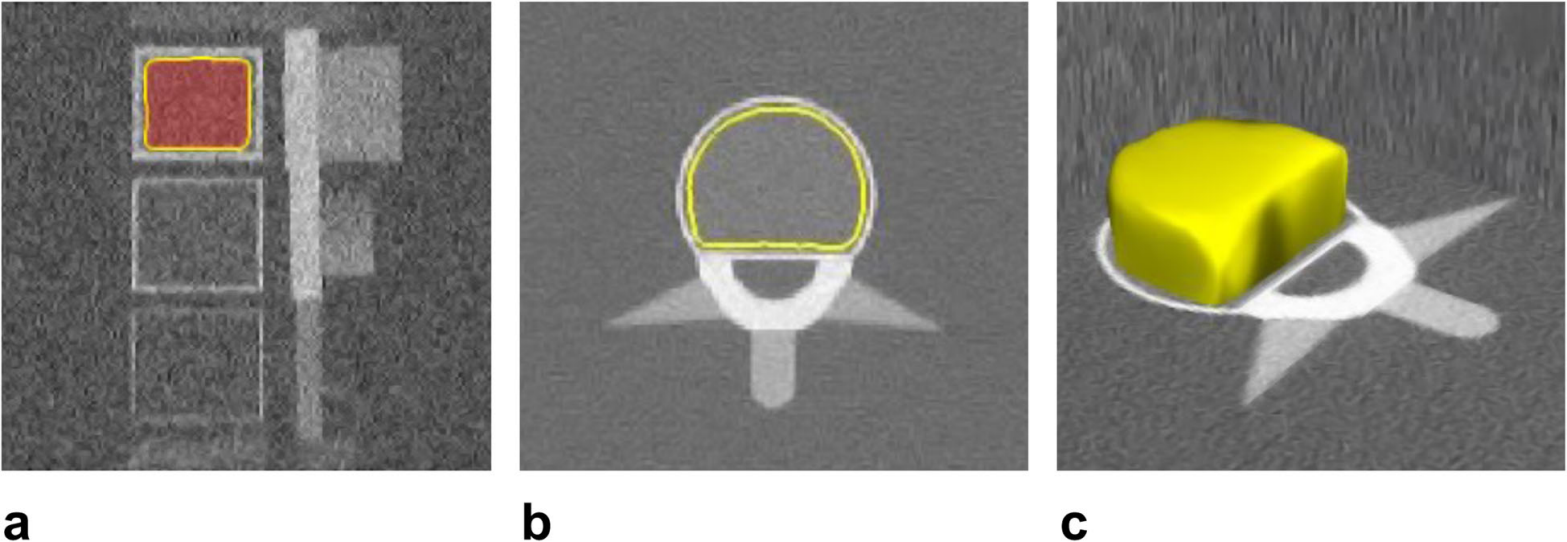
**a** The European spine phantom contains three different inserts with different nominal design concentrations of 50, 100, and 200 mg/cm^3^ HA for L1, L2, and L3, respectively. By application of specific software features for labeling of HA inserts (LiverLab; Fraunhofer Institute for Computer Graphics Research, Darmstadt, Germany), the trabecular bone (red area) was marked manually (yellow line). **b** Exemplary axial image of one HA insert showing the trabecular VOI (yellow line). **c** Three-dimensional reconstruction of the VOI which is depicted as a yellow formation. This VOI and the two DECT series (90 and Sn150 kVp) served as input for volumetric bone mineral density assessment using a second software tool (BMD Analysis; Fraunhofer Institute for Computer Graphics Research, Darmstadt, Germany), which works on material decomposition. HA, Hydroxyapatite; L, Lumbar vertebra equivalent; VOI, Volume of interest; DECT, Dual-energy computed tomography; BMD, Bone mineral density

A standardised anthropomorphic spine phantom (European spine phantom (ESP), serial number ESP-040; QRM GmbH, Moehrendorf, Germany) was used in this study (Fig. 1). The ESP represents a tool for standard quality control in DXA and QCT and contains three different hydroxyapatite (HA) inserts simulating trabecular bone densities of 50.0 (HA 50, L1), 98.4 (HA 100, L2), and 197.6 (HA 200, L3) mg/cm^3^ (Fig. 2). It should be noted that the exact HA densities as specified by the manufacturer were used for the statistical analysis, whereas the nominal values were used only for illustrational purposes in tables and figures.

For QCT calibration, a standardised QCT bone density calibration phantom (BDC, serial number BDC-03-29; QRM GmbH, Moehrendorf, Germany), which contains six cylindrical HA inserts simulating trabecular bone densities of 0, 104.4, 206.2, 402.3, 601.2, and 802.8 mg/cm^3^, was scanned together with the ESP according to clinical standard [7]. For the sake of simplicity, measurement of HA densities in both phantoms is termed BMD measurement in the remaining text. Radiation doses of each scan were noted.

### Scan protocols and image reconstruction

Scans were generated in craniocaudal direction from L1 to L3 using a third-generation dual-source DECT scanner (Somatom Force; Siemens Healthineers, Forchheim, Germany).

For QCT-based BMD measurements, the ESP and BDC phantom were examined together ten times applying the same QCT scan protocol in each scan. Images were acquired at 120 kVp and 180 mAs using an image section thickness of 1.25 mm according to our standard procedure in clinical routine [23].

For dual-source DECT-based BMD measurements, the ESP was scanned ten times using the same scan protocol as applied in our daily routine for lumbar spine imaging. The two x-ray tubes were operated at different kilovoltage settings (tube A, 90 kVp at 180 mAs; tube B, Sn150 kVp, 0.64 mm tin filter, at 180 mAs) using automatic attenuation-based tube current modulation (CARE dose 4D; Siemens Healthineers). Three image sets were created from each DECT examination: 90 kVp, Sn150 kVp, and weighted average (ratio, 0.5:0.5) for assimilating contrast properties of single-energy 120 kVp images. For volumetric BMD assessment, image series were reconstructed in axial, coronal, and sagittal planes (section thickness 0.6 mm, increment 0.6 mm) using a dual-energy bone kernel (Br69f).

DECT and QCT images were automatically transferred to the picture archiving and communication system (General Electric Healthcare GmbH, Solingen, Germany).

### Volumetric BMD assessment

QCT-derived BMD assessment was conducted according to clinical standard using a dedicated workstation and QCT software package (Mindways Software Inc., Austin, USA). Following calibration phantom scanning by using the same protocol as with ESP, the acquired calibration data was stored and used in all subsequent ESP scans for conversion of CT data into BMD values.

Phantomless DECT-based volumetric BMD assessment of L1-3 was based on material decomposition. The trabecular volume of interest (VOI) for each HA insert was manually defined by one reader (M.H.A., radiology resident with 3 years of experience in musculoskeletal imaging) using specific features of the LiverLab software package for labeling of HA inserts (LiverLab; Fraunhofer Institute for Computer Graphics Research, Darmstadt, Germany). The reader was able to determine the VOI in 3D image data sets which were generated with the uploaded DECT series and if necessary, manually modified for optimal delineation of the trabecular bone.

The obtained data of VOI and the two DECT series (90 and Sn150 kVp) were used for volumetric BMD assessment [16], which was conducted with a second software tool (BMD Analysis; Fraunhofer Institute for Computer Graphics Research) on the basis of material decomposition for each voxel, as initially described by Nickoloff et al. [10] and applied by Wichmann et al. [19]. The previously reported algorithm for material decomposition is based on a biophysical model accounting for the five major substances of trabecular bone (bone minerals, collagen matrix, water, red marrow, and adipose tissue) [21]. Using this model, the following two equations are derived:

1 $$ {X}^{90} HU=\left({\mu}^{90}-{\gamma}^{90}g\right)\cdot {V}_{TB}+\left({\beta}^{90}t-{\gamma}^{90}g\right)\cdot {V}_F+{\gamma}^{90}g+\delta $$

2 $$ {X}^{150} HU=\left({\mu}^{150}-{\gamma}^{150}g\right)\cdot {V}_{TB}+\left({\beta}^{150}t-{\gamma}^{150}g\right)\cdot {V}_F+{\gamma}^{150}g+\delta $$

These equations link the intensities *Χ*^*90*^ and *Χ*^*150*^ (given in HU) in the two CT series obtained at tube energies of 90 and 150 kV to the fraction of the volume occupied by the matrix material (bone mineral + collagen) *V*_*TB*_ and the volume of adipose tissue *V*_*F*._ The values for *t* and *g* are 0.92 g/cm^3^ and 1.02 g/cm^3^, respectively, whereas the other variables are energy related constants [10]. By calculating the mean intensity for the trabecular bone in both CT data sets, values for *V*_*TB*_ and *V*_*F*_ can be attained. Finally, the BMD value *ρ*BM (given in g/cm^3^) can be calculated from *V*_*TB*_ by application of the material constants *l* = 3.06 g/cm^3^ and *λ* = 2.11:

3 $$ \rho \mathrm{BM}=\frac{l\cdot {V}_{TB}}{1+\lambda } $$

For assessment of spatial BMD distribution, a specific BMD value for each voxel was obtained.

### Statistical analysis

Statistical analysis was performed using commercially available software (MedCalc for Windows, Version 13, MedCalc, Mariakerke, Belgium). Normality of data was tested using the Shapiro-Wilk test. Variables were illustrated as mean ± standard deviation. A *p* value of less than 0.05 indicated a statistically significant difference.

Accuracy of DECT and QCT for volumetric BMD assessment were assessed by calculating measurement errors for each HA insert. Measurement errors were defined as the difference between the measured BMD and the true BMD concentration for each HA insert as given by the manufacturer:

$$ \mathrm{Measurement}\ \mathrm{error}\;\left(\frac{\mathrm{mg}}{{\mathrm{cm}}^3}\right)=\mathrm{Measured}\;\mathrm{BMD}\kern0.3em minus\;\mathrm{True}\kern0.2em \mathrm{BMD} $$

$$ \mathrm{Relative}\ \mathrm{measurement}\ \mathrm{error}\;\left(\%\right)=\frac{\mathrm{Measurement}\kern0.17em \mathrm{error}}{\mathrm{True}\;\mathrm{BMD}}\times 100 $$

In addition, the one-sample *t* test was used to analyse both BMD assessment approaches with the true BMD values. Furthermore, the paired *t* test was used to compare values for L1, L2, and L3 on DECT and QCT. Repeatability as a parameter for precision was assessed by calculating the coefficient of variance. Correlation analysis between DECT and QCT, and between each method and the true BMD was performed by calculating Pearson product-moment correlation (*r*) and linear regression. An *r* value of less than 0.4, 0.41–0.6, 0.61–0.8, and greater than 0.8 was considered as poor, moderate, strong, and very strong, respectively. Bland-Altman plots were used to evaluate the agreement of both methods and the agreement of each method with the true BMD value.

### Results

**Fig. 3:**
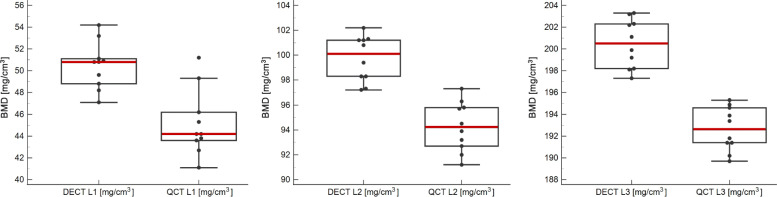
Box and whisker plots show DECT-based mean BMD values for L1 (50 mg/cm^3^), L2 (100 mg/cm^3^), and L3 (200 mg/cm^3^) of 50.47 ± 2.17 mg/cm^3^, 99.72 ± 1.84 mg/cm^3^, and 200.48 ± 2.24 mg/cm^3^, respectively; QCT-derived mean BMD values were 45.16 ± 3.04 mg/cm^3^, 94.26 ± 2.00 mg/cm^3^, and 192.66 ± 2.02 mg/cm^3^, respectively. The red line illustrates the mean and the black box edges represent the 25th and 75th percentiles. Statistical comparison resulted in significant differences between DECT and QCT for L1, L2, and L3 (all *p* < 0.001). DECT, Dual-energy computed tomography; BMD, Bone mineral density; L, Lumbar vertebra equivalent; QCT, Quantitative computed tomography

**Fig. 4:**
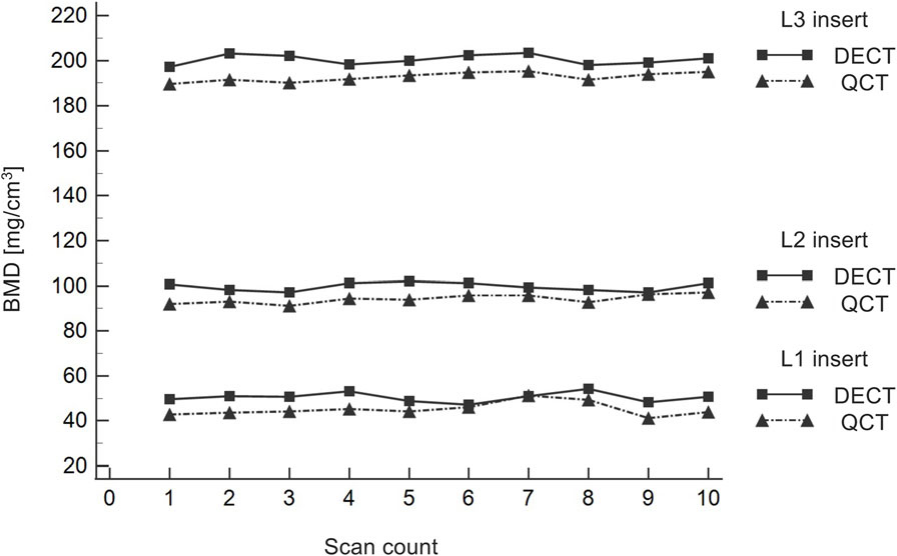
Illustration of BMD values derived from DECT and QCT for the three HA-inserts L1 (50 mg/cm3), L2 (100 mg/cm3), and L3 (200 mg/cm3). Comparisons of measured BMD values of L1, L2, and L3 with their true BMD values showed significantly lower values when using QCT (all *p* < 0.001), whereas DECT-derived BMD values differed not significantly compared to true BMD values (all *p* ≥ 0.509). BMD, Bone mineral density; DECT, Dual-energy computed tomography; QCT, Quantitative computed tomography; HA, Hydroxyapatite; L, Lumbar vertebra equivalent

Dual-source DECT-based BMD values for L1 (50 mg/cm^3^), L2 (100 mg/cm^3^), and L3 (200 mg/cm^3^) were 50.47 ± 2.17 mg/cm^3^ (mean ± standard deviation), 99.72 ± 1.84 mg/cm^3^, and 200.48 ± 2.24 mg/cm^3^, respectively; QCT-derived mean BMD values were 45.16 ± 3.04 mg/cm^3^, 94.26 ± 2.00 mg/cm^3^, and 192.66 ± 2.02 mg/cm^3^, respectively (Table 1). All data from DECT- and QCT-derived BMD measurements were normally distributed (*p ≥* 0.177). Statistical comparison resulted in significant differences between DECT and QCT for L1, L2, and L3 (all *p* < 0.001) (Fig. 3). Overall mean BMD values of L1–L3 inserts were 116.89 ± 63.53 mg/cm^3^ (range, 47.10−203.30 mg/cm^3^) for DECT, and 110.69 ± 62.42 mg/cm^3^ (range, 41.10−195.30 mg/cm^3^) for QCT (*p* < 0.001). Comparisons of measured BMD values of L1, L2, and L3 with the corresponding true BMD values showed significantly lower BMD results using QCT (all *p* < 0.001), whereas DECT-derived BMD values did not differ significantly when compared to true BMD values (all *p* ≥ 0.509) (Fig. 4).

**Table 1:** Values of bone mineral density using dual-source DECT and QCT

	L1 (50 mg/cm^3^)		L2 (100 mg/cm^3^)		L3 (200 mg/cm^3^)	
Scan	DECT	QCT	DECT	QCT	DECT	QCT
1	49.6	42.7	100.8	92.0	197.3	189.7
2	51.1	43.6	98.3	93.2	203.2	191.4
3	50.8	44.2	97.2	91.2	202.2	190.2
4	53.2	45.3	101.2	94.5	198.2	191.8
5	48.8	44.2	102.2	93.9	199.9	193.4
6	47.1	46.2	101.3	95.8	202.3	194.6
7	50.9	51.2	99.4	95.7	203.3	195.3
8	54.2	49.3	98.3	92.7	198.1	191.4
9	48.2	41.1	97.3	96.3	199.2	193.9
10	50.8	43.8	101.2	97.3	201.1	194.9
Mean ± standard deviation	50.47 ± 2.17	45.16 ± 3.04 *	99.72 ± 1.84	94.26 ± 2.00 *	200.48 ± 2.24	192.66 ± 2.02 *
Relative measurement error (%)	0.94	9.68	0.28	5.74	0.24	3.67
Absolute measurement error	0.47	4.84	0.28	5.74	0.48	7.34

Measured BMD values for L1 (50 mg/cm^3^), L2 (100 mg/cm^3^), and L3 (200 mg/cm^3^) using dual-source DECT and QCT imaging. DECT-derived BMD values were closer to true HA concentrations and differed significantly from those obtained by QCT in L1 (*p* < 0.001), L2 (*p* < 0.001), and L3 (*p* < 0.001)

*p < 0.001

*BMD* Bone mineral density, *L* Lumbar vertebra equivalent, *DECT* Dual-energy computed tomography, *QCT* Quantitative computed tomography, *HA* Hydroxyapatite

The absolute measurement errors using DECT for the L1, L2, and L3 inserts were 0.47 mg/cm^3^, 0.28 mg/cm^3^, and 0.48 mg/cm^3^, respectively, and 4.84 mg/cm^3^, 5.74 mg/cm^3^, and 7.34 mg/cm^3^ using QCT. Relative measurement errors for the L1, L2, and L3 inserts were 0.94%, 0.28%, and 0.24% (DECT), respectively, and 9.68%, 5.74%, and 3.67% using QCT (Table 1).

Repeatability of DECT-derived BMD measurements was better as compared to QCT. Coefficients of variance for the L1, L2, and L3 inserts were 4.29%, 1.85%, and 1.11% (DECT), respectively, and 6.74%, 2.12%, and 1.05% using QCT.

Mean BMD assessment time for DECT including all postprocessing steps was 4 min (range, 2−6 min), while BMD assessment took 3 min on average for QCT (range, 2−4 min).

### Correlation analysis

**Fig. 5:**
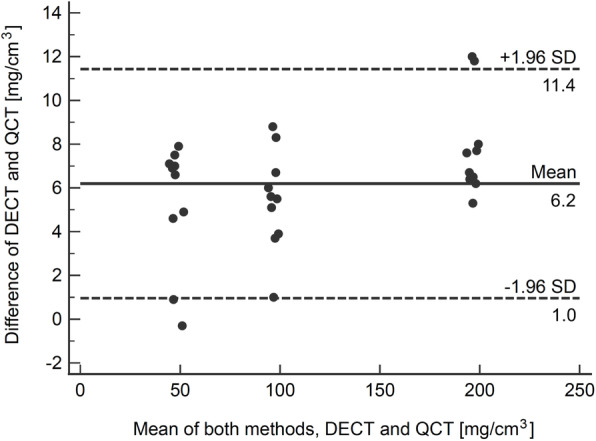
Bland-Altman plot illustrating the agreement between dual-source DECT- and QCT-based BMD measurements comprising L1 (50 mg/cm^3^), L2 (100 mg/cm^3^), and L3 (200 mg/cm^3^). Solid line: mean BMD difference. Dot line: 95% limits of agreement (mean difference ± 1.96 standard deviation). DECT, Dual-energy computed tomography; QCT, Quantitative computed tomography; BMD, Bone mineral density; L, Lumbar vertebra equivalent

Overall correlation analysis showed strong correlation between BMD values derived from DECT and QCT (*r* = 0.9993; 95% CI 0.9984–0.9997; *p* < 0.001). The corresponding Bland-Altman plot demonstrated substantial agreement between DECT and QCT (Fig. 5). Overall DECT-derived BMD values revealed a mean difference from QCT measurements of $\overline{d}$= 6.20 mg/cm^3^ (95% CI 5.20–7.19 mg/cm^3^; *p* < 0.001), with a standard deviation of 2.67 mg/cm^3^. The 95% limits of agreement were +11.43 mg/cm^3^ (95% CI 9.71–13.16 mg/cm^3^) and +0.96 mg/cm^3^ (95% CI -0.76–2.68 mg/cm^3^). DECT-derived BMD values for L1, L2, and L3 showed a mean difference from QCT of $\overline{d}$ = 5.31 mg/cm^3^ (95% CI 3.27–7.35 mg/cm^3^; *p* < 0.001), $\overline{d}$ = 5.46 mg/cm^3^ (95% CI 3.83–7.09 mg/cm^3^; *p* < 0.001), and $\overline{d}$ = 7.82 mg/cm^3^ (95% CI 6.18–9.46 mg/cm^3^; *p* < 0.001), respectively.

Furthermore, DECT- and QCT-derived BMD values were highly correlated to true BMD values (*r* = 0.9995, 95% CI 0.9989–0.9998 for DECT and *r* = 0.9993, 95% CI 0.9985–0.9997 for QCT, respectively; *p* < 0.001) without significant difference between the two correlation coefficients (*p* = 0.536).

**Fig. 6:**
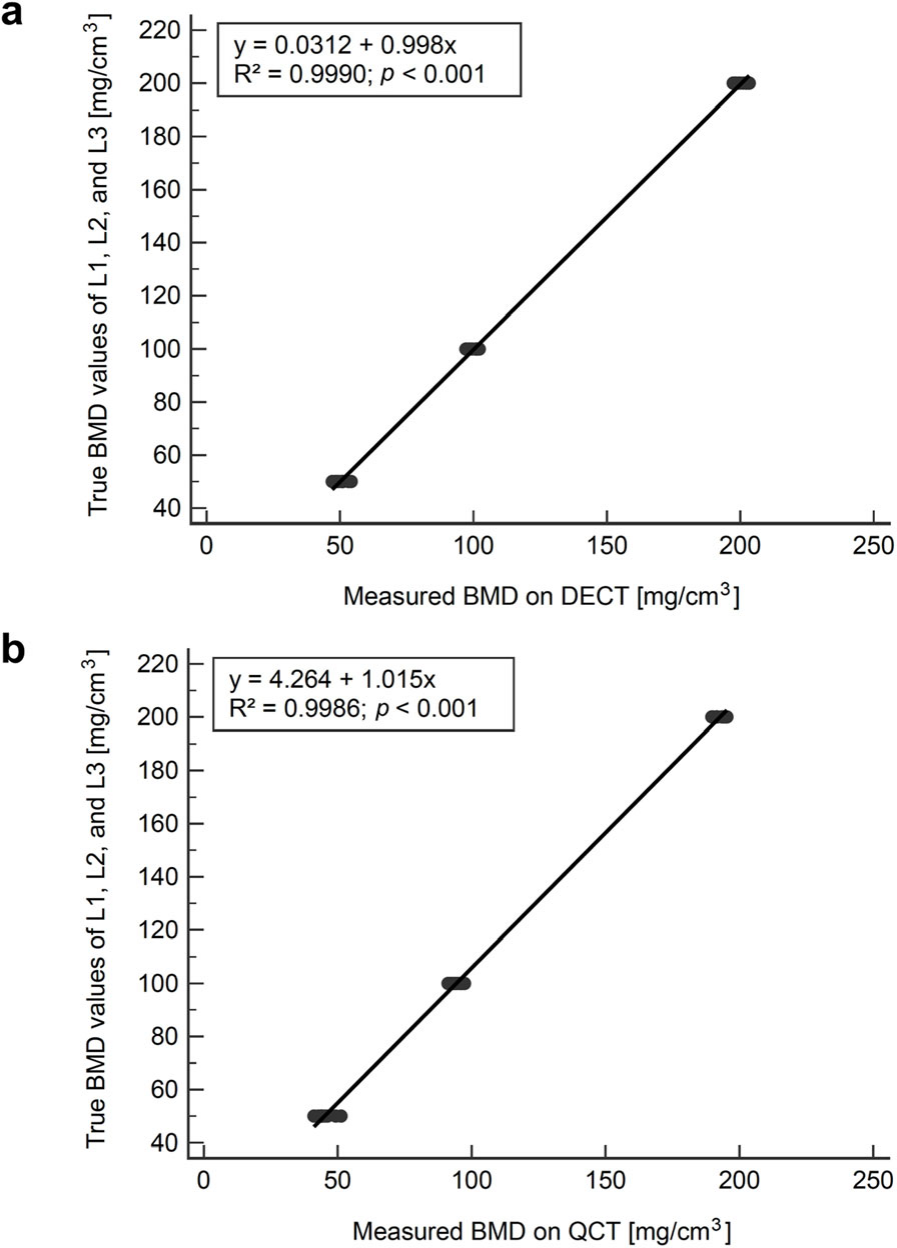
Linear regression of BMD values in (**a**) DECT and (**b**) QCT. Scatter plots showing the relationship between true BMD values of L1, L2, and L3 (*y*-axis) and measured BMD values obtained by DECT (**a**) or QCT (**b**) (*x*-axis); the black lines represent the linear regression lines. A significant correlation between true and measured BMD values was identified, both in DECT (*R*^*2*^ = 0.9990; *p* < 0.001) and QCT (*R*^*2*^ = 0.9986; *p* < 0.001). BMD, Bone mineral density; DECT, Dual-energy computed tomography; QCT, Quantitative computed tomography; L, Lumbar vertebra equivalent; R^2^, Coefficient of determination

Linear regression analysis was performed on DECT- and QCT-based BMD values for scaling of values and reduction of deviations. For DECT, the regression equation was *y* = 0.0312 + 0.998x with *R*^2^ = 0.9990 (Fig. 6a). Using this equation, BMD values for L1, L2, and L3 were scaled to 50.40 ± 2.16 mg/cm^3^, 99.55 ± 1.84 mg/cm^3^, and 200.11 ± 2.23 mg/cm^3^, respectively. The relative measurement errors were adjusted to 0.80%, 0.45%, and 0.06%, respectively.

For QCT, the regression equation was *y* = 4.264+1.015x with *R*^2^ = 0.9986 (Fig. 6b). Applying this equation, BMD values for L1, L2, and L3 were scaled to 50.10 ± 3.07 mg/cm^3^, 99.94 ± 2.03 mg/cm^3^, and 199.81 ± 2.04 mg/cm^3^, respectively. The relative measurement errors were reduced to 0.20%, 0.06%, and 0.10%, respectively.

### Radiation dose

The values of CTDI_vol_ values comprising the ten CT scans were 9.89 ± 0.33 mGy (mean ± standard deviation) (range, 9.50–10.40 mGy) and 12.41 ± 0.27 mGy (range, 11.90–12.70 mGy) for DECT and QCT (*p* < 0.001), respectively. Mean dose-length product (DLP) values were 198.54 ± 6.82 mGy × cm (range, 190.40–209.10 mGy × cm) for DECT and 219.18 ± 8.69 mGy × cm (range, 205.60–234.80 mGy × cm) for QCT (*p* < 0.001) (Table 2).

**Table 2:** Radiation doses

	CTDI_vol_ (mGy)	DLP (mGy × cm)	kVp	mAs
DECT	9.89 ± 0.33	198.54 ± 6.82	Tube A, 90; tube B, Sn150	180
QCT	12.41 ± 0.27	219.18 ± 8.69	120	180

Mean CTDI_vol_ and DLP values differed significantly between DECT and QCT (*p* < 0.001). Data are given as mean ± standard deviation

*CTDI*_*vol*_ Volumetric CT dose index, *DLP* Dose length product, *DECT* Dual-energy computed tomography, *QCT* Quantitative computed tomography

## Discussion

Our study showed that phantomless volumetric dual-source DECT-based BMD assessment performed on a spine phantom with three different known HA concentrations yields higher accuracy and precision in comparison to QCT, the current clinical standard for volumetric BMD assessment. In this context, DECT-derived BMD measurements showed a maximal relative measurement error of 0.94% in comparison to 9.68% (QCT). Repeatability of BMD measurement was better using DECT as compared to QCT (coefficient of variance for DECT < 4.29% and for QCT < 6.74%). DECT-derived spine phantom measurements demonstrated high agreement and correlation with QCT (*r* = 0.9993; 95% CI 0.9984–0.9997; *p* < 0.001). The mean BMD assessment time using DECT was 4 min indicating time-efficient applicability in clinical routine.

Opportunistic assessment of BMD has increasingly attracted scientific attention along with demographic changes and the growing prevalence of osteoporosis among older adults [1, 2]. Considering the fact that more than 80 million CT scans are performed each year in the USA [24], patients may highly profit from accurate and precise automated BMD measurements which can be easily derived from routine CT examinations, resulting in lowered costs, resources, radiation exposure, and in a more time-efficient clinical workflow [25]. In this context, application of HU measurements for BMD assessment have been proposed [14, 15, 26], despite being associated with known inaccuracies. Based on the contribution of photoelectric and Compton interactions, bone attenuation values are profoundly affected by tube voltage levels, and the inhomogeneity of trabecular bone composition further influences HU measurements [13, 27]. In addition, the wide range of different devices and manufacturers as well as the applied technique might also affect BMD assessment based on HU measurements.

Nickoloff et al. [10] developed a dedicated material decomposition model based on DECT which facilitates phantomless volumetric opportunistic BMD assessment of trabecular bone. Based on this model, a dual-source DECT postprocessing algorithm for volumetric phantomless BMD assessment of the lumbar spine was introduced [16]. Wichmann et al. [19] demonstrated the feasibility of this approach in clinical routine, and further evaluated cancellous DECT-based BMD assessment of thoracic and lumbar pedicles in a cadaver study [20]. In addition, a recently published study has shown that phantomless volumetric BMD assessment based on dual-source DECT yields superior diagnostic accuracy for the detection of osteoporosis compared to HU measurements [21]. However, to the best of our knowledge, no prior study has compared dual-source DECT and QCT for volumetric BMD assessment of lumbar vertebrae with regards to accuracy and precision.

Available DECT techniques include dual-source, rapid kilovoltage switching, and dual-layer spectral technologies [28–30]. Dual-source DECT and rapid kilovoltage switching DECT represent the current market leading technologies [28]. Li et al. demonstrated in a recently published phantom study that BMD can be accurately measured either by using DECT or QCT with even smaller bias using DECT compared to QCT [31]. Relative measurement errors of the three different HA inserts were comparable to our study, both in DECT and QCT. The DECT method for BMD assessment was based on rapid kilovoltage switching (Revolution CT, GE, Waukesha, WI, USA) using a different material decomposition algorithm and postprocessing software compared to dual-source DECT. The published phantom study results based on rapid kilovoltage switching were recently reproduced *in vivo* [32]. BMD quantification of the lumbar spine from in total 128 consecutive patients showed a strong correlation and agreement between DECT- and QCT-derived BMD measurements (*R*^*2*^ = 0.983–0.987). However, it should be noted that the applied technique of rapid kilovoltage switching suffers from known limitations regarding modulation and filtration of tube current which can affect BMD measurements, and acquisition times are longer when compared to dual-source DECT [28]. The dual-layer detector technique operates by application of a superficial and a deep layer. Known limitations of this technology include relatively high radiation dose and reduced soft tissue contrast preventing its widespread clinical use in comparison to dual-source DECT [28]. Nevertheless, there have been several studies evaluating the accuracy of dual-layer spectral CT for BMD assessment. In this context, Hamersvelt et al. [33] demonstrated the possibility of accurate BMD quantification using dual-layer spectral CT with strong linear correlations (*R*^*2*^ ≥ 0.970; *p* < 0.001) to DXA. Additionally, Roski et al. [34] found high correlations between BMD values derived from dual-layer spectral CT and those from QCT by analysing 174 vertebrae in 33 patients. Another study on the feasibility of dual-layer CT-derived BMD assessment in vertebral specimens and phantom-calibrated QCT measurements using different setups with varying degrees of obesity reported high correlation between the two modalities unaffected by the obesity grade [35].

In accordance with the aforementioned studies using fat-free phantoms, our observations demonstrated higher BMD values in almost all CT scans obtained with dual-source DECT in comparison to QCT at simultaneously reduced measurement errors. A possible reason for this discrepancy could be technical features of the new generation of DECT spectral imaging technology who comes along with many advantages resulting in better image quality. However, the underestimation of QCT-derived BMD is well known in the clinical setting and may partly be explained by the fat error which significantly affects density measurements and can potentially result in overdiagnosis of osteoporosis [34, 36]. In this context, QCT is influenced by the variable amount of marrow fat leading to accuracy errors of QCT measurements ranging from 2 to 30% [26, 37, 38]. Reasons for this include the operating principle of the single-energy technique, which can only analyse a volume of two components (for example bone mass and red marrow), not taking marrow fat into consideration [37]. Due to dedicated material decomposition, fat-related inaccuracies of BMD measurement can be minimised and overcome by DECT [39].

Another major limitation of QCT-derived BMD assessment represents the need for calibration phantoms that do not represent the true composition of trabecular bone, eliminating the option for opportunistic BMD assessment in routinely performed CT scans which are increasingly conducted in DECT mode. In contrast, DECT offers retrospective phantomless BMD measurements resulting in greater flexibility in clinical routine, rendering additional DXA and QCT examinations unnecessary and thereby substantially reducing radiation exposure, particularly in young patients, premenopausal women, and patients with chronic diseases undergoing repeated follow-up CT scans [19]. While the radiation dose of DXA is relatively low with an effective dose of 0.013 mSv in adults, protocols using QCT for BMD evaluation report effective doses between 1 and 3 mSv [40]. Radiation dose in our study differed significantly between DECT and QCT (*p* < 0.001). Mean CTDI_vol_ values for QCT were similar to the data provided by Li et al., whereas CTDI_vol_ values for DECT were lower [31]. In addition, DECT has the potential to reduce metal artifacts and permit BMD assessment surrounding metallic implants in the context of adjacent segment degeneration and an increased risk for postoperative fractures [41]. Finally, dual-source DECT-based retrospective BMD assessment may also influence spinal surgeries. The known potential of this technique to compute segmental BMD assessment and to enable a colour-coded three-dimensional display of trabecular BMD distribution might improve preoperative planning of spondylodesis and ensure placement of the pedicle screw in a vertebral segment with higher stability [42].

This study has certain limitations that need to be addressed. First, we used a phantom setting without considering confounding factors that affect BMD assessment, such as the use of intravenous contrast medium, age, gender, body mass index, presence of metal implants, and the fat fraction within vertebral bone marrow. The influence of these factors on BMD assessment could not be evaluated in our phantom study and should be considered in future *in vivo* research. Second, dual-source DECT was evaluated using a single scan protocol, which represents the standard protocol for CT-based lumbar spine imaging in our department. Thus, results and conclusions are vendor- and protocol-specific. Nevertheless, this setup facilitated systematic evaluation of a routinely performed scan protocol under controlled conditions. Third, the ESP phantom represents a relatively small adult person. It thus remains unclear whether our results and conclusions are transferable to patients with other constitutions. Fourth, the accuracy of our DECT approach on calculating volumetric BMD should be evaluated using other energy spectra. Fifth, only predefined lumbar vertebra equivalents were examined without considering biologic variability of BMD and possible BMD variations throughout the lumbar spine [11, 43]. Finally, results of our phantom study have to be confirmed in future studies on humans, evaluating the reproducibility of BMD measurements.

In conclusion, our phantom study demonstrated that phantomless dual-source DECT-derived material decomposition allows for more accurate volumetric BMD assessment compared to QCT at significantly lower radiation dose. Therefore, opportunistic dual-source DECT-derived BMD assessment may serve as a viable alternative to QCT, avoiding unnecessary radiation exposure.

## Data Availability

All data generated or analysed during this study are included in this published article.

## Abbreviations

BMD: Bone mineral density
CI: Confidence interval
CT: Computed tomography
CTDIvol: Volumetric CT dose index
DECT: Dual-energy CT
DLP: Dose length product
DXA: Dual-energy x-ray absorptiometry
ESP: European spine phantom
HA: Hydroxyapatite
HU: Hounsfield units
QCT: Quantitative CT
VOI: Volume of interest
